# Success to Clear the Bar in Elite Pole Vaulters is Affected by Step Frequency Perturbation

**DOI:** 10.5114/jhk/163060

**Published:** 2023-07-15

**Authors:** Apostolos S. Theodorou, Vassilios Panoutsakopoulos, Timothy A. Exell, Johan Cassirame, Hervé Sanchez, Mariana C. Kotzamanidou

**Affiliations:** 1 School of Physical Education & Sport Science (SEFAA), National and Kapodistrian University of Athens, Dafni Athens, Greece.; 2 Biomechanics Laboratory, School of Physical Education and Sport Science at Thessaloniki, Aristotle University of Thessaloniki, Thessaloniki, Greece.; 3 School of Sport, Health and Exercise Science, University of Portsmouth, Portsmouth, UK.; 4 EA 4660, Department Sport et Performance, Université de Franche-Comté, Laboratoire C3S, Besançon, France.; 5 EA 7507, Laboratoire Performance, Santé, Métrologie, Société, Universite de Reims Champagne Ardenne, Reims, France.; 6 FFA – French Athletics Federation, Recherche Division, Paris, France.; 7 Mtraining, R&D Division, École Valentin, France.; 8 Faculty of Health Sciences, Metropolitan College of Thessaloniki, Thessaloniki, Greece.

**Keywords:** kinematics, laterality, inter-limb asymmetry, performance, approach run

## Abstract

The purpose of the study was to identify the interaction of step characteristics, along with the direction and magnitude of their asymmetry of elite male and female pole vaulters between successful and failed attempts. It was hypothesized that step characteristics and the magnitude of asymmetry between the two legs would interact with the outcome of the attempt. The approach runs of 12 pole vaulters (7 males, 5 females) were recorded during an indoor international competition. The leg used by the athlete for taking-off was defined as the non-pole-carrying leg, while the other was the pole-carrying leg. Using spatiotemporal information obtained from recordings with a panning camera (300 fps), the last steps of each athlete's approach run were analyzed for length, frequency, average velocity, and inter-limb asymmetry. There was no inter-limb difference (p > 0.05) in the absolute values of step length or step velocity between successful and failed attempts. However, the pole-carrying leg presented significantly (p < 0.05) higher step frequency values at the failed attempts. There was no significant difference (p > 0.05) in asymmetry values for step length, frequency, and average velocity between successful and failed attempts. Although step velocity remained unaffected, failed attempts were characterized by a perturbation in the interaction of step frequency and step length. The present findings suggest that although high velocity at the final phase of the approach is essential, it is not the sole determining factor for a successful attempt.

## Introduction

In pole vault competition, the athlete’s horizontal velocity during the last steps appears to serve as a good indicator of the vaulter’s potential performance and significant correlation is reported between run-up velocities and jump height for both men and women (Adamczewski and Perlt 1997; Angulo-Kinzler et al., 1994; Arikawa et al., 2016; Cassirame et al., 2017; Choi et al., 2013). Greater approach velocity enables the athlete to use a higher grip and, therefore, larger kinetic energy to be transmitted on the pole while planting it in the take-off box (Angulo-Kinzler et al., 1994; Frère et al., 2010). Comparison between successful and failed conditions for the same vaulters showed trivial and small differences in the individual approach profiles, but a greater speed at the take-off for successful compared to failed attempts (Cassirame et al., 2019). However, exceptions to this pattern have also been shown with vaulters of both genders managing to improve their performance with a little increase in approach velocity (Adamczewski and Perlt, 1997).

Step velocity (SV) is directly affected by step length (SL) and step frequency (SF) (Salo et al., 2011). Maintaining consistency in these variables ensures that the athlete’s spatiotemporal kinematics are replicated across multiple attempts. Observations on long jumpers (Theodorou et al., 2017) revealed that during the last phase of the approach, velocity was increased due to the increment in SF rather than SL. Similarly, at the plant preparation phase, the “gravity-assisted” drop of the pole facilitates an increase in SF to a greater extent than in SL (Gros and Kunkel, 1990).

Bilateral asymmetry and the possible predominance or preference of a limb for regulating velocity during the approach run can also play a role in SL and SF interactions. Asymmetry has recently been the focus of attention in the literature for its part in the biomechanics of sprint running (Exell et al., 2012a, 2012b), the horizontal jumps approach (Theodorou et al., 2017), and the pole vault approach (Panoutsakopoulos et al., 2021). Theodorou et al. (2017) reported individual asymmetry profiles for SL and SF in long jumpers favoring a different limb for each step variable, but eventually resulting in no significant asymmetry in SV. Pole vault is also an asymmetrical jumping event, as significant inter-limb asymmetries in SF were observed in the last steps of the approach (Panoutsakopoulos et al., 2021). Carrying the pole has a slightly higher magnitude effect on exerted force than velocity, which suggests a higher force deficit that affects horizontal thrust (Frère et al., 2017). During the acceleration phase, vaulters run with the pole fixed at a particular position and are forced to lean backward to counter its weight, adversely affecting sprinting mechanics due to the created anterior imbalance (Cassirame et al., 2022; Frère et al., 2009). Subsequently, at the final phase of the approach, the lowering of the pole increases gravitational torque forcing the centre of gravity of the vaulter-pole system to translate ahead of ground support. This action reduces the time the swing leg needs to recover in a normal sprinting position and sufficiently accelerate the foot posteriorly before contacting the ground (Frère et al., 2017). Additionally, the unilateral position and carriage of the pole leads to asymmetrical arm positioning that restricts both arms from swinging. This constraint on arm motion reduces longitudinal shoulder rotations and affects leg coordination and hip rotations (Novacheck, 1998). However, despite this constraint, vaulters, just like long jumpers, do not present asymmetry for SV probably through the unconscious manipulation of the inter-limb interaction between SL and SF (Theodorou et al., 2017).

Based on these observations, it is probable that any slight deviation in the interaction pattern of step characteristics that occurs during a jump due to a change in task constraints (i.e., bar height, pole stiffness) could lead to a failed attempt if other factors remain the same. The aim of the study was to determine step characteristic interaction as well as the direction and magnitude of their asymmetry between successful and failed attempts during a major competition. It was hypothesized that step characteristics and the magnitude of asymmetry between the two legs would interact with the outcome of the attempt (successful/failed). The results of the present study could assist coaches in identifying the patterns of SV determinants that lead to a successful bar clearance.

## Methods

### Participants

Data from the performances of seven male (age 26.3 ± 3.4 years, body height 1.84 ± 0.06 m, body mass 73.0 ± 7.6 kg, performance 5.81 ± 0.09 m) and five female (age 25.9 ± 4.2 years, body height 1.68 ± 0.03 m, body mass 58.6 ± 4.8 kg, performance 4.71 ± 0.12 m) elite pole vaulters were collected during the finals of the 2017 Indoor European Athletics Championship in Belgrade. Approval for the investigation was granted by the Organizing Committee and the Institutional Research Committee of the School of Physical Education and Sport Sciences (SEFAA), National and Kapodistrian University of Athens, Athens, Greece (approval no.: 1002/23-2-2017). Participating athletes provided informed consent.

### Measures

**Figure 1:**
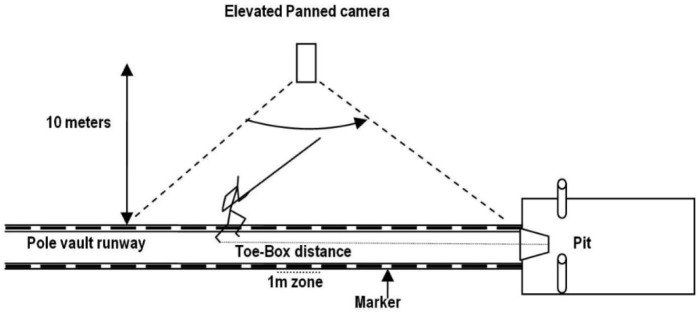
Schematic illustration of the experimental setup.

A manually panned high-speed camera (Casio EX F1; Casio Computer Co. Ltd., Shibuya, Japan) recorded the final phase of the approach at a sampling rate of 300 fps and a shutter speed of 1/1000 s. Markers were placed at 1 m intervals on the runway’s lines. The camera was placed near the spectator seats, about 15 m from the centre line of the track and at a height of about 3 m (Figure 1).

The toe-box distance (the space between footfall and the back edge of the plant box) was calculated according to the method suggested by Hay and Koh (1988) and validated as described by Theodorou et al. (2013). Finally, details about the pole lengths used by participants were also collected. A simple electronic web questionnaire was constructed and distributed among participants to gain information about the pole characteristics (length, stiffness) that was used at each attempt.

### Design and Procedures

Attempts selected for further analysis for each vaulter were the pair of jumps including a failed attempt to clear a particular height and a successful subsequent jump at the same height. Another selection criterion for the selected pair of jumps was that the length of the approach and pole properties were the same (Cassirame et al., 2019). Using the above inclusion criteria, the late approach phase of 17 pairs of jumps where vaulters had a failed and a successful jump at the same height (*n* = 34) was selected for analysis. The height cleared at the selected jumps ranged from 4.45 to 5.85 m. All other attempts that did not fulfil the above inclusion criteria were excluded from the analysis.

The frames containing the instance of foot contact on the surface at each of the last 8 steps of the approach were analysed using the APAS Wizard v.13.3.0.3 software (Ariel Dynamics, Inc., Trabuco Canyon, CA, United States). The last step of the approach run was omitted from the analysis since it is not representative in terms of running kinematics (Makaruk et al., 2016).

Toe-box distance (TBD) was defined as the horizontal distance between the vaulter’s toe and the back frame of the plant box. SL was calculated by deducting two consecutive toe-box distances. Step time (tS) was defined as the elapsed time from one-foot’s last contact on the ground to the opposite foot’s last contact on the ground. SV of each step was evaluated with equation 1, while SF was calculated using equation 2:


$$SV=\frac{SL}{t_{s}}\;\left(1\right)$$

$$SF=\frac{1}{t_{s}}\;\left(2\right)$$

Individual step parameter asymmetry was calculated according to the method described by Exell et al. (2012a). The take-off leg was defined as the non-pole-carrying (NPC) leg, while the other as the pole-carrying (PC) leg. Symmetry angle (*ϑ_SYM_*) values for steps following the PC foot take-off and steps following the NPC foot take-off were calculated according to the equation 3 proposed by Zifchock et al. (2008):


$$\vartheta_{SYM}=\frac{\left(45{}^{\circ}-\arctan\left(\frac{x_{NPC}}{x_{PC}}\right)\right)}{90{}^{\circ}}\times 100\%\;\left(3\right)$$

where θ_SYM_ is the symmetry angle, X_PC_ is the mean value for the PC step and X_NPC_ is the value for the NPC step. However, if:


$$\left(45{}^{\circ}-\arctan\left(\frac{x_{NPC}}{x_{PC}}\right)\right)>90{}^{\circ}$$

then equation 3 was substituted with equation 4:


$$\vartheta_{SYM}=\frac{\left(45{}^{\circ}-\arctan\left(\frac{x_{NPC}}{x_{PC}}\right)-180{}^{\circ}\right)}{90{}^{\circ}}\times 100\%\;\left(4\right)$$

The significance (*p* < 0.05) of asymmetry between PC and NPC values for each step characteristic in terms of intra-limb variability (Exell et al., 2012b) was examined (following Shapiro-Wilk tests for normality) with Mann-Whitney U tests.

### Statistical Analysis

The homogeneity of variances and the normality of distribution were established following the Levene’s (*p* > 0.05) and the Kolmogorov-Smirnov (*p* > 0.05) tests, respectively. A 2 (result: failed/successful) x 2 (leg: NPC/PC) ANOVA with Bonferroni adjustments was used to determine the main effect of result and leg and their interaction with SF, SL, and SV values. Effect sizes were examined with the partial eta-squared statistic (*η_p_^2^*). Additionally, an independent samples T-test was used to determine possible differences concerning θ_SYM_ for SF, SL, and SV between successful and failed attempts. Effect sizes were examined using Cohen’s *d*. In all statistical analyses the level of significance was set at *α* = 0.05.

## Results

For SF, the leg (*p* < 0.05, large effect size) but not the result (*p* > 0.05) main effect was significant. No significant leg and result main effects were observed for SL and SV (Table 1). The interaction effect of result and leg was not significant for SL, SF, and SV. Concerning θ_SYM_, no significant difference (*p* > 0.05) due to the result was observed for SL, SF, and SV (Table 2).

**Table 1:** Descriptive statistics (Mean ± *SD*), main effects and interaction of result and leg for step length (SL), frequency (SF), and velocity (SV).

Variable	Leg	Successful	Failed			
		(Mean ± *SD*)	(Mean ± *SD*)	*F*	*p*	*η*_p_^2^
SL (m)	PC	2.10 ± 0.18	2.08 ± 0.20	**Result effect**		
	NPC	2.12 ± 0.19	2.14 ± 0.19	0.001	0.981	0.000
				**Leg effect**		
				0.895	0.348	0.014
				**Interaction**		
				0.167	0.684	0.003
SF (Hz)	PC	4.19 ± 0.21*	4.22 ± 0.24*	**Result effect**		
	NPC	4.03 ± 0.15	4.02 ± 0.15	0.036	0.850	0.001
				**Leg effect**		
				15.132	< 0.001*	0.191
				**Interaction**		
				0.294	0.590	0.005
SV (m/s)	PC	8.76 ± 0.61	8.75 ± 0.68	**Result effect**		
	NPC	8.55 ± 0.64	8.59 ± 0.60	0.010	0.920	0.000
				**Leg effect**		
				1.515	0.223	0.023
				**Interaction**		
				0.034	0.854	0.001

NOTE: PC: pole carrying leg; NPC: non-pole carrying leg; * p < 0.05

**Table 2:** Descriptive statistics (Mean ± *SD*) and successful vs. failed comparison for the symmetry angle (θ_SYM_) of step length (SL), frequency (SF), and velocity (SV).

Result	SUCCESSFUL (*n* = 34)	FAILED (*n* = 34)	*t*	*p*	*d*
**SL θ_SYM_ (°)**	0.90 ± 0.69	1.34 ± 1.15	1.355	0.185	0.46
**SF θ_SYM_ (°)**	1.41 ± 0.99	1.86 ± 1.38	1.105	0.278	0.38
**SV θ_SYM_ (°)**	1.06 ± 0.85	1.08 ± 0.86	0.048	0.962	0.02

## Discussion

The purpose of the study was to improve understanding regarding the interaction between step characteristics, and their asymmetry when comparing successful and failed attempts in elite pole vaulters. Among the two step variables that determine the speed of the athlete, SL was the one with the greater consistency. Increasing SF while maintaining the same SL is considered a prerequisite for a successful pole plant and a practice advocated by coaches (Gross et al., 2020). According to the evidence published so far, it is still unclear if this induces a reduced ability for greater horizontal force production due to pole carriage (Plessa et al., 2010) or the necessity for an effective vertical impulse and a decreased duty factor (Weyand et al., 2000) rather than extended swing time for transferring energy onto the pole. Furthermore, at the late phase of the approach, greater precision and consistency in SL is in effect due to the visual steering mechanism compared with the acceleration phase, where most of the foot placement variability occurs (Cassirame et al., 2019). According to Theodorou et al. (2017), the increase in SV at the end of the approach run of high-level long jumpers was induced by a 5% increase in SL and a 13% increase in SF from the one established at the acceleration phase. The balanced contribution of SL to the development of SV at the late approach phase was confirmed in the present study by the lack of differences between attempts or legs.

On the other hand, SF was not similar in both legs, but was higher in the PC leg. The last phase of the approach is characterized by an active pole drop, a technique that allows athletes to exploit the kinetic energy of the drop for increasing their velocity right into the take-off (Gros and Kunkel, 1990). It is likely that the change in the pole position from vertical to horizontal and the subsequent increase in gravitational torque in front of the PC leg’s foot during its ground contact create conditions for particular unilateral assisted sprinting. It is interesting though that this increase in SF of the PC leg compared with the NPC leg was significantly greater at the failed attempts. The question that arises is why a much higher increase in SF of the PC leg was observed in the failed attempts. Potential causes for this difference in unsuccessful attempts could be the actions that occur at the pole carrying side. A smooth and continuous pole drop allows a good posture and is used as a method to increase velocity and acceleration of the approaching take-off. Conversely, if the pole drop is less smooth, the torque of the pole will increase the load on the athlete's body (Yang et al., 2021). It could be that, at the failed attempts, athletes dropped the pole too fast, an action that caused the athlete’s trunk to be pulled forward and was counteracted by shortening their step, which eventually led to a further increase in the vertical impulse and SF turnover of the PC leg.

Although this increase in SF of the PC leg per se may not be undesirable, it may prove detrimental if it leads to the asymmetry of step characteristics. Velocity is maximized by emphasizing either SL, by exploiting the forward impulse, or SF, by diminishing contact time and increasing the vertical impulse (Debaere et al., 2013). The lack of asymmetry in the resultant SV suggests that this increase in SF was probably offset by a respective increase in SL, as advocated by previous research (Theodorou et al., 2017). The increased SL symmetry angle values at the failed attempts in the present study, although not statistically significant, support this assumption. Consequently, this perturbation of the interaction of SF and SL, although with no effect on velocity, may have contributed to the failed attempt by affecting the positional requirements at the take-off, the variability of the take-off foot (Needham et al., 2018), or the vertical impulse required for transferring energy onto the pole (Angulo-Kinzler et al., 1994).

The data of the web questionnaire revealed that participants opted not to change the length of the pole, but to use a pole of the same length for their attempts at the same height. Thus, vaulters might have changed their pattern of regulation of the step characteristics and consequently, may have contributed to the failed attempt. This poses a limitation in the current study since step regulation was not assessed. Nevertheless, previous research (Needham et al., 2018) suggested that high-level pole vaulters could adapt their running patterns and that step regulation in pole vault did not affect the technical execution of the vault (Frère et al., 2019). Another possible limitation is the examination of both sexes in a single group with a limited number of participants. Based on findings of the relative literature, the examination of the attempts in terms of the outcome and not sex is justified, as the temporal and relative spatial variables seem not to differ between elite male and female vaulters (Hanley et al., 2022; Panoutsakopoulos et al., 2021).

## Conclusions

The present study revealed that there was no difference in the absolute values of SL or SV between successful and failed attempts. However, during the failed attempts, the SF of the PC leg was significantly greater than the respective values of the NPC leg. Additionally, no significant difference in symmetry values was recorded between failed and successful attempts for SV. Nevertheless, there was a trend for increased symmetry angle values of SF and SL at the failed attempts. This finding indicates a perturbation in the interaction of these two step characteristics that may be responsible for an unsuccessful outcome.

In conclusion, the present findings support the notion that a speedy approach alone is not the only determining factor for a successful jump. Coaches should aim to foster stable patterns considering the athlete-pole interaction by giving emphasis on stable pole drop patterns and step contacts in the final phase of the approach during their training routines. Further sources of individual variability in the interaction of SF and SL could also be examined in their conflicting requirements, such as differences in the moments of inertia of the legs, their explosive strength, and the effect of the pole drop.
